# A Rapid, Simple, Inexpensive, and Mobile Colorimetric Assay COVID-19-LAMP for Mass On-Site Screening of COVID-19

**DOI:** 10.3390/ijms21155380

**Published:** 2020-07-29

**Authors:** Franklin Wang-Ngai Chow, Tony Tat-Yin Chan, Anthony Raymond Tam, Suhui Zhao, Weiming Yao, Joshua Fung, Flora Ka-Kei Cheng, George Chi-Shing Lo, Stella Chu, Kam Leng Aw-Yong, James Yat-Man Tang, Chi-Ching Tsang, Hayes Kam-Hei Luk, Antonio Cheuk-Pui Wong, Kenneth Sze-Ming Li, Longchao Zhu, Zirong He, Emily Wan Ting Tam, Tom Wai-Hin Chung, Sally Cheuk Ying Wong, Tak-Lun Que, Kitty Sau-Chun Fung, David Christopher Lung, Alan Ka-Lun Wu, Ivan Fan-Ngai Hung, Patrick Chiu-Yat Woo, Susanna Kar-Pui Lau

**Affiliations:** 1 Department of Microbiology, Li Ka Shing Faculty of Medicine, The University of Hong Kong, Hong Kong 19/F, Block T, Queen Mary Hospital, 102 Pokfulam Road, Hong Kong; cwn5810@gmail.com (F.W.-N.C.); tonytychan1@gmail.com (T.T.-Y.C.); suhui@connect.hku.hk (S.Z.); yao719082506@126.com (W.Y.); joshua6061@gmail.com (J.F.); florakkc@gmail.com (F.K.-K.C.); gcslo@connect.hku.hk (G.C.-S.L.); stellaachuu@gmail.com (S.C.); kamleng@hotmail.com (K.L.A.-Y.); jamesymtang@connect.hku.hk (J.Y.-M.T.); microbioct@connect.hku.hk (C.-C.T.); hayesluk@gmail.com (H.K.-H.L.); antoniow.nycoris@gmail.com (A.C.-P.W.); keth105@gmail.com (K.S.-M.L.); longchao@connect.hku.hk (L.Z.); hezirong725@hotmail.com (Z.H.);; 2 Department of Medicine, Queen Mary Hospital, Hong Kong 102 Pok Fu Lam Road, Pok Fu Lam, Hong Kong Island, Hong Kong; antamwf@connect.hku.hk; 3 Department of Food and Health Sciences, Faculty of Science and Technology, Technological and Higher Education Institute of Hong Kong, Hong Kong Tsing Yi Road, Tsing Yi, Hong Kong; emilytam@vtc.edu.hk; 4 Department of Microbiology, The University of Hong Kong, Queen Mary Hospital, Hong Kong Block T, Queen Mary Hospital, 102 Pokfulam Road, Hong Kong; cwh366@ha.org.hk; 5 Department of Pathology, Queen Elizabeth Hospital, Hong Kong 10/F, Block M, Queen Elizabeth Hospital, 30 Gascoigne Road, Hong Kong; wcy288@ha.org.hk (S.C.Y.W.); h9910303@yahoo.com.hk (D.C.L.); 6 Department of Pathology, Tuen Mun Hospital, Hong Kong Pathology Block, 23 Tsing Chung Koon Road, Tuen Mun, New Territories, Hong Kong; quetl@ha.org.hk; 7 Department of Pathology, United Christian Hospital, Hong Kong 130 Hip Wo Street, Kwun Tong, Hong Kong; fungsck@ha.org.hk; 8 Department of Clinical Pathology, Pamela Youde Nethersole Eastern Hospital, Hong Kong 3 Lok Man Road, Chai Wan, Hong Kong; alanklwu@yahoo.com; 9 Department of Medicine, Li Ka Shing Faculty of Medicine, The University of Hong Kong, Hong Kong 4/F, Professorial Block, Queen Mary Hospital, 102 Pok Fu Lam Road, Hong Kong; ivanhung@hku.hk; 10 State Key Laboratory of Emerging Infectious Diseases, The University of Hong Kong, Pokfulam, Hong Kong Laboratory Block, 21 Sassoon Road, Pokfulam and University Pathology Building, Queen Mary Hospital, The University of Hong Kong, Hong Kong

**Keywords:** COVID-19, RT-LAMP, SARS-CoV-2, mass screening, on-site screening, diagnosis, mobile Diagnostic

## Abstract

To control the COVID-19 pandemic and prevent its resurgence in areas preparing for a return of economic activities, a method for a rapid, simple, and inexpensive point-of-care diagnosis and mass screening is urgently needed. We developed and evaluated a one-step colorimetric reverse-transcriptional loop-mediated isothermal amplification assay (COVID-19-LAMP) for detection of SARS-CoV-2, using SARS-CoV-2 isolate and respiratory samples from patients with COVID-19 (*n* = 223) and other respiratory virus infections (*n* = 143). The assay involves simple equipment and techniques and low cost, without the need for expensive qPCR machines, and the result, indicated by color change, is easily interpreted by naked eyes. COVID-19-LAMP can detect SARS-CoV-2 RNA with detection limit of 42 copies/reaction. Of 223 respiratory samples positive for SARS-CoV-2 by qRT-PCR, 212 and 219 were positive by COVID-19-LAMP at 60 and 90 min (sensitivities of 95.07% and 98.21%) respectively, with the highest sensitivities among nasopharyngeal swabs (96.88% and 98.96%), compared to sputum/deep throat saliva samples (94.03% and 97.02%), and throat swab samples (93.33% and 98.33%). None of the 143 samples with other respiratory viruses were positive by COVID-19-LAMP, showing 100% specificity. Samples with higher viral load showed shorter detection time, some as early as 30 min. This inexpensive, highly sensitive and specific COVID-19-LAMP assay can be useful for rapid deployment as mobile diagnostic units to resource-limiting areas for point-of-care diagnosis, and for unlimited high-throughput mass screening at borders to reduce cross-regional transmission.

## 1. Introduction

The novel coronavirus disease 2019 (COVID-19), caused by the novel human severe acute respiratory syndrome coronavirus 2 (SARS-CoV-2), has become a global pandemic with enormous economic impact [1,2]. SARS-CoV-2 is a member of subgenus *Sarbecovirus* under genus *Betacoronavirus* of the family *Coronaviridae*, being closely related to human and bat severe acute respiratory syndrome coronaviruses (SARS-CoVs) of the species *Severe acute respiratory syndrome-related coronavirus* [3,4,5,6]. In contrast to SARS-CoV and Middle East respiratory syndrome coronavirus (MERS-CoV), which were rapidly traced to civet and dromedaries, respectively, as the source of the epidemics [7], the origin of SARS-CoV-2 remains obscure [8]. Rapid and accurate identification and segregation of SARS-CoV-2 infected patients including asymptomatic viral shedders are critical to reduce human-to-human transmission and disease impact [9].

The current gold standard for molecular diagnosis of COVID-19 is based on the detection of SARS-CoV-2 RNA by real-time quantitative reverse transcription-polymerase chain reaction (qRT-PCR) [10,11,12,13,14,15]. Although qRT-PCR is highly sensitive and specific, it requires expensive qPCR instruments and experienced laboratory technologists to perform the assay and interpret test results. As such, clinical samples often need to be transported to a qualified laboratory at a distance. Therefore, the resulting cost and turn-around-time of qRT-PCR renders its application infeasible for mass detection of SARS-CoV-2 within a short time, especially for low-income countries or in remote areas where hospitals may not be equipped with molecular diagnostic laboratories. Although portable assays or qPCR machines are also potentially useful for rapid detection of SARS-CoV-2, they are very expensive and only a few samples can be processed at a time [16,17]. For example, only one sample can be tested by the Abbott ID NOW which costs US$ > 10,000 for the machine and US$ > 100 for each sample (Table 1). As for enzyme-linked immunosorbent assays for antigen/antibody detection, they are often insensitive and non-specific. Moreover, positive antibodies are only detected after 7–14 days and cannot differentiate between acute and past infection. Therefore, they are not useful for identifying actively infected COVID-19 patients.

**Figure 1:**
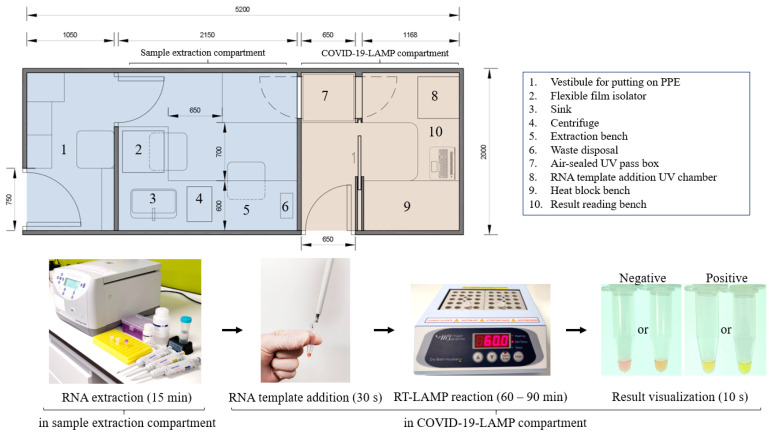
Illustration of a small van-sized mobile COVID-19-LAMP diagnostic unit. A drawn-to-scale layout of a van-sized mobile COVID-19-LAMP diagnostic unit, with sample processing and LAMP reactions compartments have been illustrated. A cargo van/lorry can be modified quickly to become a mobile diagnostic unit for rapid deployment in any region.

Here, we report the development and evaluation of a rapid and simple one-step colorimetric reverse transcriptional loop-mediated isothermal amplification (RT-LAMP) assay, COVID-19-LAMP, for detection of SARS-CoV-2. RT-LAMP combines reverse transcriptase, DNA polymerase, pH indictor, and six primers to amplify RNA templates [18,19,20,21,22,23,24], causing a drop in pH and, thus, a color change from pink to yellow. Since RT-LAMP involves both reverse transcription and DNA amplification at a constant temperature without the need for a PCR thermal cycler [25], it only requires simple equipment, namely benchtop centrifuges, heat blocks, and micropipettes. The skills involved are easy to master by junior laboratory technologists or healthcare workers with same-day training within hours. Evaluation using clinical samples from COVID-19 patients and patients with other respiratory virus infections showed that this COVID-19-LAMP assay is highly sensitive and specific. The result can be unambiguously visualized by the naked eye and interpreted by any person, with short turn-around-time (including sample extraction) of 45–105 min depending on the viral load (Figure 1).

## 2. Results

### 2.1. COVID-19-LAMP Assay Can Detect SARS-CoV-2 with a Low Detection Limit

LAMP primer set targeting a region across orf3a and E gene of SARS-CoV-2 was able to amplify the targeting region, as confirmed by Sanger sequencing, and color changes were observed within reaction tubes. The optimal reaction temperature and primer concentrations were determined at 60 °C, outer primer (F3, B3: 0.18 μM), inner primer (FIP, BIP: 0.73 μM), and loop primer (LoopF, LoopB: 0.36 μM) (Supplementary Table S1). Using RNA extracted from SARS-CoV-2 isolate, the assay had a limit of detection of 42.0 copies per reaction. The results indicate that this COVID-19-LAMP is potentially sensitive for detection of SARS-CoV-2.

### 2.2. COVID-19-LAMP Assay Is Highly Sensitive and Specific for Detection of SARS-CoV-2 in Clinical Samples

Of all 223 respiratory samples positive for SARS-CoV-2 by qRT-PCR, 212 and 219 samples were tested positive by COVID-19-LAMP assay at 60 and 90 min, showing sensitivities of 95.07% (95% CI: 0.92–0.98) and 98.21% (95% CI: 0.96–1.00) respectively (Table 2). The highest sensitivity was observed among nasopharyngeal swabs, with 93 and 95 of 96 samples positive by RT-LAMP at 60 and 90 min, showing sensitivities of 96.88% and 98.96%, respectively. For sputum/deep throat saliva samples, 63 and 65 of 67 samples were positive at 60 and 90 min, showing sensitivities of 94.03% and 97.02%, respectively. For throat swab samples, 56 and 59 of 60 samples were positive at 60 and 90 min, showing sensitivities of 93.33% and 98.33%, respectively. None of the 143 samples with other respiratory viruses were positive by RT-LAMP at 90 min, showing 100% specificity for all sample types (Table 3).

**Figure 2:**
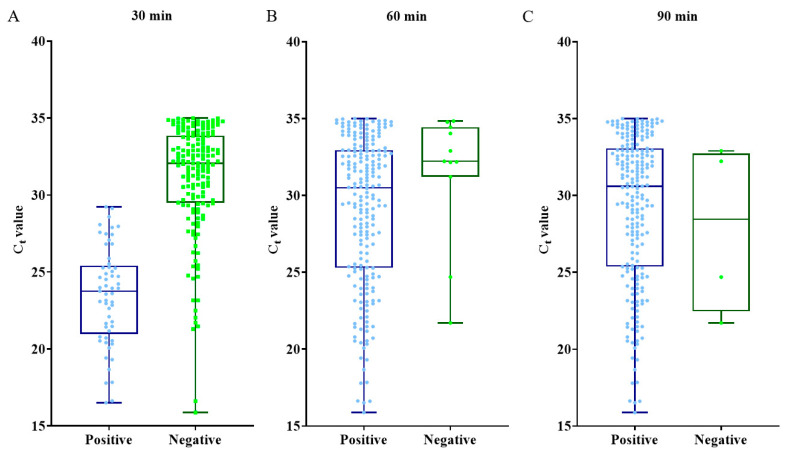
Box and whisker plot of COVID-19-LAMP results. The COVID-19-LAMP results of each SARS-CoV-2 qRT-PCR positive samples have been illustrated in the box and whisker plot at (**A**): 30 min, (**B**): 60 min, (**C**): 90 min with corresponding qRT-PCR C_t_ values of samples.

Samples positive by COVID-19-LAMP assays showed C_t_ values between 15.88 to 35.00 by qRT-PCR. There was positive correlation between time to positivity by COVID-19-LAMP (four levels: 30 min, 60 min, 90 min, >90 min), and qRT-PCR C_t_ values, with Spearman’s rank order correlation coefficient of 0.63 (*p* < 0.0001), suggesting that samples with higher viral loads turned positive at an earlier time (Figure 2).

## 3. Discussion

This simple, highly sensitive and specific COVID-19-LAMP assay would be very useful for implementation into ad hoc diagnostic units for rapid deployment in any locations, allowing point-of-care diagnosis on one hand and mass screening for guiding public health measures on the other. The assay showed high sensitivities for detection of SARS-CoV-2 among tested respiratory samples, especially for nasopharyngeal swabs with 96.88% and 98.96% sensitivity at 60 and 90 min, respectively, compared to qRT-PCR assay as gold standard. COVID-19-LAMP failed to detect four qRT-PCR-positive samples; it could be due to the carryover of inhibitors from the extraction process. Moreover, the specificity was 100%, with no false positive even among samples with other human coronaviruses. Samples with higher viral loads took shorter time to turn positive. Positive results were recorded as early as 30 min for samples with higher viral loads (mean ± SD C_t_ value 23.47 ± 3.19) (Figure 2), although the highest sensitivity was observed at 90 min. This assay is also highly cost-effective, with reagents for each reaction costing only US$2–4, compared to US$20–60 for qRT-PCR assay (excluding the cost for sample extraction of US$5–8 and one heat block for 48–96 samples of US$100–1000) (Table 1). Moreover, it is a portable assay requiring only simple equipment and techniques, without the need for installing expensive qPCR machines or employing experienced molecular biologists. It also saves the cost, time, and risks of transporting samples to laboratories especially during the lockdown of cities.

This COVID-19-LAMP assay can be deployed as mobile diagnostic units such as in vehicles to communities with limited access to major hospitals or laboratories, allowing rapid point-of-care diagnosis at private or regional clinics or hospitals with suspected COVID-19 cases. An example of a small van-sized unit is depicted in Figure 1. This is particularly useful to high-incidence or less developed countries, where the capacity for qRT-PCR assays cannot meet the test demand. These RT-LAMP units can also be mobilized to quarantine camps or facilities to test residents developing COVID-19 symptoms and upon release to document viral clearance. It is estimated that two to three workers will be enough in a diagnostic unit to handle about hundreds of samples per day. Widespread application of this RT-LAMP assay can help speed up diagnosis and treatment to reduce mortality and facilitate infection control measures to reduce disease transmission of this pandemic virus in badly affected countries.

Since this assay only involves simple laboratory procedures and can be scaled up easily, hundreds or thousands of samples can be processed at the same time, allowing high-throughput rapid on-site mass screening at quarantined areas and borders with results available in less than two hours. For example, travelers departing or arriving by air can be screened at airport COVID-19-LAMP diagnostic centers and those tested positive can be immediately sent to hospitals or isolation facilities. Diagnostic units can also be set up easily at cruise terminals, train stations, or highway control points. Such a mass-screening strategy can help minimize disease import/export between high- and low-incidence areas. This is of particular benefit to areas where community outbreaks are relatively under control, such as Hong Kong, Taiwan, Australia, and New Zealand, etc., where mass screening at the borders can allow rapid identification and isolation of infected returners from high disease impact countries, avoiding new massive outbreaks. Those returners tested negative can then be allowed to enter the cities after a reasonable testing time. When the high-incidence areas succeed to bring down the case numbers dramatically, this assay can be used at the borders to avoid re-importing new source of infections and hence subsequent epidemic waves. Until the availability of an effective vaccine for SARS-CoV-2, the world will have to adopt strict international traffic control and universal personal hygiene measures. This COVID-19-LAMP can serve as a cost-effective method for mass screening, allowing social activities and the economy to function at its maximum.

Concerning biosafety, in places where biosafety cabinet is not available for sample processing before virus inactivation, portable flexible film isolator can be used as an alternative with full personal protective equipment with N95 respirators in a separately ventilated compartment is recommended to prevent cross contamination of samples and laboratory-acquired infection. The use of a flexible film isolator allows performing extraction under negative pressure environment and filtering with HEPA filter. Disinfectant agents, such as Virkon, can be used for disinfection inside the flexible film isolator after sample extraction to prevent any carryovers. Regular maintenance and fumigation of the flexible film isolator can also be carried out to ensure the function and safety of the isolator. Since the virus in clinical samples is rapidly inactivated by the lysis buffer at the first step of nucleic acid extraction, all subsequent procedures can be safely performed with biosafety level 1 (BSL-1) practice, with appropriate settings to avoid RNA or LAMP amplicon contamination which may result in false-positive reactions. Another concern with this colorimetric assay is that it requires the recognition of color change by naked eye. Though not difficult, generally, the results can only be interpreted by workers without color blindness. One possible solution is to use a colorimeter or a smartphone colorimeter application which can be used for objective result interpretation.

## 4. Materials and Methods

### 4.1. Patient Samples

Respiratory samples collected from 40 hospitalized patients with laboratory-confirmed COVID-19, including four asymptomatic patients and other respiratory virus infections in Hong Kong were included in this study. The collection and use of clinical samples and data were approved by the Institutional Review Board of the University of Hong Kong/Hospital Authority Hong Kong West Cluster (UW 16-365 20-07-2016).

### 4.2. SARS-CoV-2 Culture

Viral culture of SARS-CoV-2 was performed in biosafety level-3 (BSL-3) facility. SARS-CoV-2 was isolated from a COVID-19 patient in our locality. SARS-CoV-2 was propagated in Vero cells in minimum essential medium (MEM) (Gibco, Waltham, MA, USA) supplemented with 1% fetal bovine serum (FBS) (Thermo Fisher Scientific, Waltham, MA, USA). Vero cells were seeded onto 24-well tissue culture plates (NEST, Wuxi, China), with 1 mL of MEM with 10% FBS, at 1 × 10^6^ cells/mL in 24 wells plate and incubated at 37 °C in a 5% carbon dioxide (CO_2_) incubator for 24 h until >90% confluence (~0.2 × 10^6^ cells). Vero cells were washed once with phosphate-buffered saline (PBS) (Oxoid, Waltham, MA, USA) and inoculated with three multiplicity of infection (MOI) of SARS-CoV-2 in serum-free MEM, and then incubated at 37 °C. After 1 h of infection, unbound virus was removed by washing with 1 mL of PBS twice. The infected cells were maintained in MEM with 1% FBS until virus-induced cytopathic effect (CPE) was observed after two days. The supernatant was collected and was spun down at 2000× *g* to remove cell debris for subsequent RNA extraction.

### 4.3. RNA Extraction from SARS-CoV-2 Culture Supernatant and Respiratory Samples

Cell culture supernatant and respiratory samples were subjected to RNA extraction by QIAamp Viral RNA Mini kit (QIAGEN, Hilden, Germany) according to manufacturer’s instructions. Briefly, RNA from 140 μL of each specimen was extracted and eluted with 60 μL of AVE buffer.

### 4.4. Development of COVID-19-LAMP Assay

Six LAMP primers were designed manually to target a region across orf3a and Envelope (E) genes of SARS-CoV-2 (Supplementary Table S1 and Supplementary Figure S2). The optimal RT-LAMP reaction conditions, including primer concentrations (outer primer F3, B3: 0.18–0.042 μM; inner primer FIP, BIP: 1.45–0.3 μM; LoopF, LoopB: 0.36–0.073 μM), temperature (50–70 °C) and reaction time (30 min, 60 min, 90 min), were determined using SARS-CoV-2 RNA extracted from culture supernatant of infected Vero cells (Supplementary Figure S1) on a Proflex Thermal Cycler (Thermo Fisher Scientific, Waltham, USA). Each 25 μL colorimetric RT-LAMP reaction contained 12.5 μL WarmStart Colorimetric LAMP 2× Master Mix (New England Biolabs, Ipswich, MA, USA), 7.5 μL mixture of outer primer (F3, B3: 0.18 μM), inner primer (FIP, BIP: 0.73 μM), and loop primer (LoopF, LoopB: 0.36 μM), and 5 μL RNA template.

The optimal conditions determined were then used for COVID-19-LAMP assay on clinical samples as described above, except that heat block instead of thermal cycler was used for RT-LAMP reaction at 60 °C. All reaction tubes were spun down before visualization of results to avoid condensation of solution. A change in color from pink to yellow or amber indicated a positive reaction, while pink or coral pink color was regarded as a negative reaction (Figure 1). A recommended workflow and standard operating procedure of this COVID-19-LAMP is provided in the Supplementary Materials (Supplement S1).

To determine the detection limit, RNA extracted from SARS-CoV-2 culture supernatant were tested by COVID-19-LAMP and quantified by N1 Probe of 2019-nCoV CDC qPCR probe assay (IDT, Coralville, IA, USA) with Superscript III Platinum One-Step qRT-PCR kit (Thermo Fisher Scientific, Waltham, USA) in a LightCycler 480 real-time PCR system (Roche, Risch-Rotkreuz, Switzerland). The copy number for limit of detection determination was calculated by using 2019-nCoV_N_Positive Control (IDT, Coralville, USA). Each 20 μL reaction mixture contained 10 μL of 2 × Superscript III Platinum Master Mix, 1.5 μL of SARS-CoV-2 CDC qPCR N1 Probe, 1 μL of SuperScript III RT/Platinum Taq Mix and 5 μL RNA templates. The one-step qRT-PCR condition used was as follows: (1) reverse transcription for 15 min at 50 °C, (2) pre-denaturation for 2 min at 95 °C, and (3) 45 cycles of denaturation for 3 s at 95 °C, and annealing and elongation for 30 s at 55 °C.

### 4.5. Evaluation of COVID-19-LAMP Assay Using Clinical Samples

**Figure 3:**
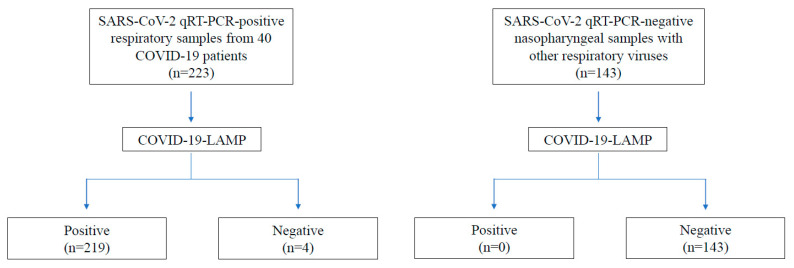
COVID-19-LAMP evaluation profile. SARS-CoV-2 qRT-PCR positive and negative respiratory samples are used for evaluation of COVID-19-LAMP.

A total of 223 respiratory samples positive for SARS-CoV-2 by qRT-PCR, including 96 nasopharyngeal swab, 67 sputum/deep throat saliva, and 60 throat swab samples from 40 COVID-19 patients hospitalized in our locality, including four asymptomatic patients; and 143 nasopharyngeal samples positive for other respiratory viruses including human enteroviruses (A71, D68 and coxsackievirus A6), adenovirus, parainfluenza viruses (1, 2, and 3), influenza viruses (A and B), respiratory syncytial virus, human metapneumovirus, human rhinoviruses (A, B, and C), human coronaviruses (OC43, 229E, HKU1, and NL63), were subject to COVID-19-LAMP assay (Figure 3). The results were interpreted with the naked eye, by two independent persons who were unaware of SARS-CoV-2 qRT-PCR results. There were no discrepancies between the two readers. The diagnostic sensitives and specificities were calculated using the web-based tool “Diagnostic Statistics” (https://www2.ccrb.cuhk.edu.hk/stat/ConfidenceInterval.htm). Box and whisker plots were generated and Spearman’s correlation was performed to study the correlation between time to positivity by COVID-19-LAMP and qRT-PCR C_t_ values by using GraphPad Prism version 8.14 (GraphPad Software, San Diego, CA, USA).

## Figures and Tables

**Table 1:** Comparison of existing COVID-19 molecular diagnostic tests with COVID-19-LAMP.

	qRT-PCR	Portable qRT-PCR	Automated Platform	Point-of-Care Diagnostic Machine	COVID-19-LAMP
**Timing**	75–90 min	75–90 min	60–120 min	15–20 min	30–90 min
**User requirement**	Experienced lab technologist		Junior lab technologist or healthcare worker with short training		
**Reagent cost/test (USD)**	$20–60	$20–60	>$110	>$100	$2–4
**Equipment cost**	>$45,000	>$4500	>$32,000	>$10,000	$100–1000
**Apparatus**	qRT-PCR machine	Portable qRT-PCR machine	GeneXpert/Filmarray	Abbott ID NOW	Heat block
**RNA Extraction**	Required		Not required		Required
**Capacity**	96 samples per run	16 samples per run	Up to 16 samples per run	1 sample per run	Unrestricted (48–96 samples per block)
**Point-of-care testing**	Not feasible	Not feasible	Feasible	Feasible	Feasible
**Mass on-site screening**	Partially feasible	Not feasible	Not feasible	Not feasible	Feasible
**Result readout**	Not easy	Not easy	Easy	Easy	Easy
	(C_t_ value may require interpretation		(report by machine)		(naked eye)

**Table 2:** Evaluation of COVID-19-LAMP using respiratory samples confirmed positive for SARS-CoV-2 by qRT-PCR (*n* = 223).

Reaction Time	Number of Positives	Number of Negatives	Sensitivity (95% CI)
**Total Respiratory Samples Positive for SARS-CoV-2 by qRT-PCR (*n* = 223)**			
60 min	212	11	95.07% (0.92–0.98)
90 min	219	4	98.21% (0.96–1.00)
**Nasopharyngeal Swabs Positive for SARS-CoV-2 by qRT-PCR (*n* = 96)**			
60 min	93	3	96.88% (0.93–1.00)
90 min	95	1	98.96% (0.97–1.00)
**Sputum/Deep Throat Saliva Positive for SARS-CoV-2 by qRT-PCR (*n* = 67)**			
60 min	63	4	94.03% (0.88–1.00)
90 min	65	2	97.02% (0.93–1.00)
**Throat Swabs Positive for SARS-CoV-2 by qRT-PCR (*n* = 60)**			
60 min	56	4	93.33% (0.87–1.00)
90 min	59	1	98.33% (0.95–1.00)

**Table 3:** Absence of cross-reactivity of COVID-19-LAMP with other human respiratory viruses.

Respiratory Samples with Other Respiratory Viruses	Number Tested	COVID-19-LAMP
Parainfluenza virus 1	10	Negative
Parainfluenza virus 2	10	Negative
Parainfluenza virus 3	10	Negative
Influenza A virus	20	Negative
Influenza B virus	6	Negative
Adenovirus	18	Negative
Respiratory syncytial virus	20	Negative
Human metapneumovirus	2	Negative
Human rhinovirus A	3	Negative
Human rhinovirus B	3	Negative
Human rhinovirus C	3	Negative
Human enterovirus A71	7	Negative
Human enterovirus D68	10	Negative
Coxsackievirus A6	10	Negative
Human coronavirus HKU1	5	Negative
Human coronavirus NL63	1	Negative
Human coronavirus 229E	1	Negative
Human coronavirus OC43	5	Negative

## Supplementary Materials

Supplementary materials can be found at https://www.mdpi.com/1422-0067/21/15/5380/s1.
