# Supplementary material for: A Rapid, Simple, Inexpensive, and Mobile Colorimetric Assay COVID-19-LAMP for Mass On-Site Screening of COVID-19
Source: Int J Mol Sci. 2020 Jul 29;21(15):5380. doi: 10.3390/ijms21155380 (PMC7432162; doi:10.3390/ijms21155380)
Supplement: Supplementary file 1 [file ijms-21-05380-s001.pdf]

Supplementary Materials

Supplementary Table S1. RT-LAMP primers for SARS-CoV-2 detection

	Primer sequence (5'-3')[a]	Position[b]	Concentration[c]	Temperature
F3	CAAATWCACACAATCGACG	6-24	0.18 μM	60℃
B3	TTAACAATATTGCAGCAGTACGCAC	244-268	0.18 μM	
FIP	GAAACGAATGAGTACATAAGTTCGTATGATGARCCGACGACGACTACTA	118-142 , 64-87	0.73 μM	
BIP	AGGTACGTTAATAGTTAATAGCGTAAATCGAAGCGCAGTAAGGATGGCTA	152-176 , 217-241	0.73 μM	
LoopF	CTTGTGCTTACAAAGGCACGCTA	86-108	0.36 μM	
LoopB	TTGCTTTYGTGGTATTCTTGCTA	187-209	0.36 μM	

[a] W represents A/T; R represents G/A; Y represents C/T
[b] SARS-CoV-2 isolate Wuhan-Hu-1 (GenBank accession no. NC_045512.1) was used as the reference genome for designing the primers.
[c] Optimal primer concentrations are given in micromole per litre (μM) based on the final reaction mix.

Supplementary Figure S1. Development of COVID-19-LAMP assay.

A. Optimization of COVID-19-LAMP reaction temperatures with RNA from SARS-CoV-2 isolates (632.5 viral copies per reaction).
From left to right, 50°C, 52.5°C, 55°C, 57.5°C, 60°C, 62.5°C, 65°C, 67.5°C, 70°C.
B. Limit of Detection (LOD) test for COVID-19-LAMP with RNA from SARS-CoV-2 isolates. From left to right, 632.5, 76.7, 42.0, 14.6, 7.3 viral copies per reaction.

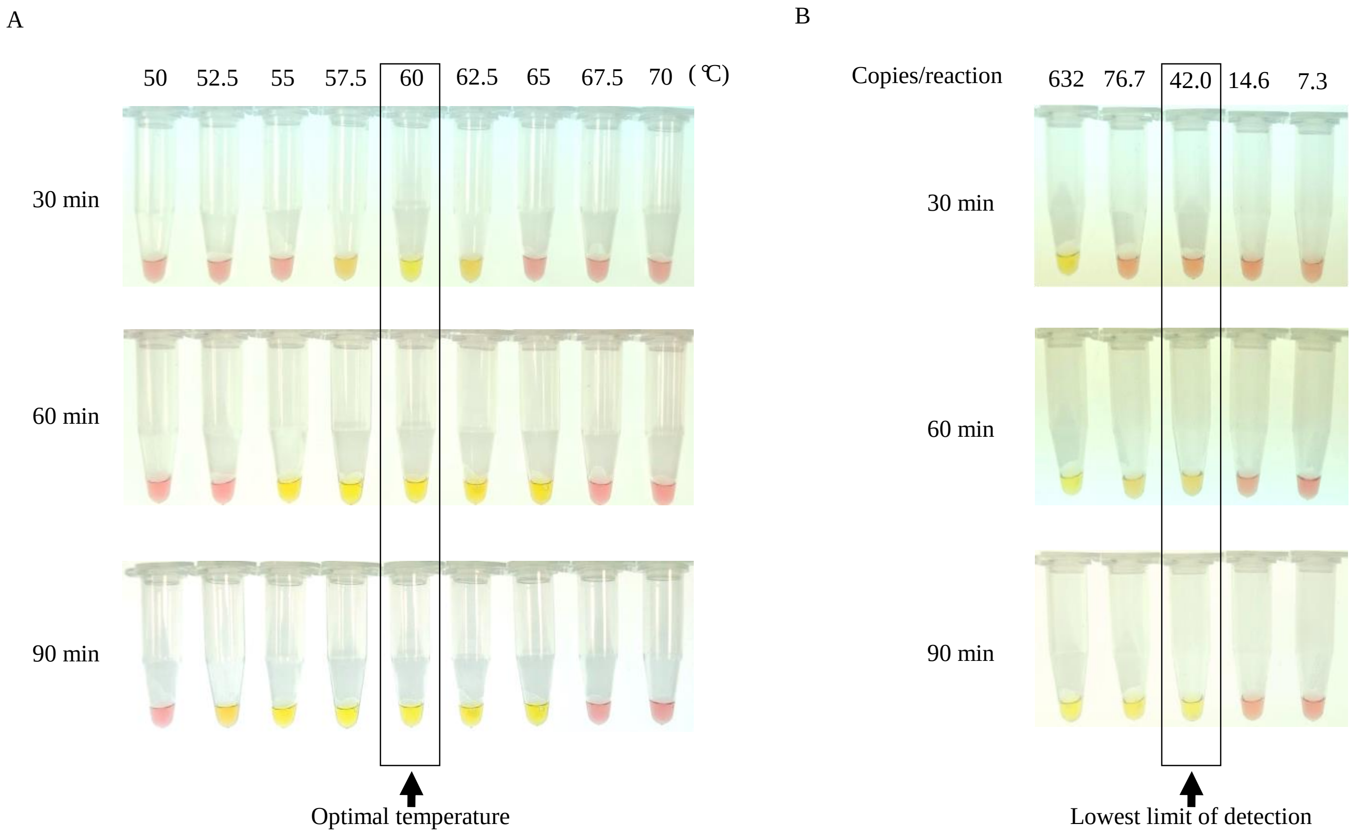

Supplementary Figure S2. Sequence alignment for COVID-19-LAMP primers design.

Sequence alignment of SARS-CoV-2 (NC_045512), HCoV 229E (NC_002645), HCoV HKU1 (NC_006577), MERS-CoV (NC_019843), HCoV OC43 (NC_006213), HCoV NL63 (NC_005831), SARS-CoV (FJ959407) for RT-LAMP primer design targeting region of orf3a and E gene. The underlined sequence indicated the primer binding site: F3 (6-24), B3 (244-268), F1c (118-142), F2 (64-87), B1c (152-176), B2 (217-241), LoopF (86-108), LoopB (187-209).

```
                            ....|....| ....|....| ....|....| ....|....| ....|....|
                                    10         20         30         40         50
NC_045512_SARS-CoV-2        ATGTCCAAAT TCACACAATC GACGGTTCAT CCGGAGTTGT TAATCCAGTA
NC_002645_HCoV 229E         ATGTTC--CT TAAGCTAGTG GATGATCATG CTTTGGTTGT TAATGTACTA
NC_006577_HCoV HKU1         CAGTTCCTCT TCACATAATC GCC------- CCGAGCTCGC TTATCGTTTA
NC_019843_MERS-CoV          CAGTTCCTCT TCACATAATC GCC------- CCGAGCTCGC TTATCGTTTA
NC_006213_HCoV OC43         ATAAATATTT TGGAGTAATA AATGGTTTCA CAGCATTCGC TAATACTGTA
NC_005831_HCoV NL63         AGGTTCTTGT GAAAAGAACC TACAACTTAT GCGTAAGGTT GACTTGTATA
FJ959407_SARS-CoV           ATGTGCAAAT ACACACAATC GACGGCTCTT CAGGAGTTGC TAATCCAGCA

                            ....|....| ....|....| ....|....| ....|....| ....|....|
                                    60         70         80         90        100
NC_045512_SARS-CoV-2        A-TGGAACCA ATTTATGATG AACCGACGAC GACTACTAGC GTGCCTTTGT
NC_002645_HCoV 229E         C-T----CTG GTGTGTGGTG CTTATAGTGA TACTACTAGT GTGTATTACA
NC_006577_HCoV HKU1         AGCAGCTCTG CGCTACTATG GGTCCCGTGT AGAGGCTAAT CCATTAGTCT
NC_019843_MERS-CoV          AGCAGCTCTG CGCTACTATG GGTCCCGTGT AGAGGCTAAT CCATTAGTCT
NC_006213_HCoV OC43         G-AGGATGCT GTTAACAAAC TGGTTTTCTT AGCTGTTGAC TT--TATTAC
NC_005831_HCoV NL63         A-TGGTGCTG TCATTTACAT TTTTGCCGAA GAGC------ ---CTGTTGT
FJ959407_SARS-CoV           A-TGGATCCA ATTTATGATG AGCCGACGAC GACTACTAGC GTGCCTTTGT

                            ....|....| ....|....| ....|....| ....|....| ....|....|
                                   110        120        130        140        150
NC_045512_SARS-CoV-2        AAGCACAAGC TGATGAGTAC GAACTTATG- ---TACTCAT TCGTTTCGGA
NC_002645_HCoV 229E         ATAATTAAAC TAATTAAGCT TTGTTTCAC- ---TTGCCAT ATGTTTTGTA
NC_006577_HCoV HKU1         CTCTTTGGAC ATATGGAAAA CGAACTATG- ---TTACCCT TTGTCCAAGA
NC_019843_MERS-CoV          CTCTTTGGAC ATATGGAAAA CGAACTATG- ---TTACCCT TTGTCCAAGA
NC_006213_HCoV OC43         CTGGCGCAGA CAGGAGTTAA ATGTTTATG- ---GCTGATG CTTATCTTGC
NC_005831_HCoV NL63         TGGTATAGTC TACTCTTCTC AACTATACGA AGATGTTCCT TCGATTAATT
FJ959407_SARS-CoV           AAGCACAAGA AAGTGAGTAC GAACTTATG- ---TACTCAT TCGTTTCGGA

                            ....|....| ....|....| ....|....| ....|....| ....|....|
                                   160        170        180        190        200
NC_045512_SARS-CoV-2        AGAG------ --ACAGGTAC GTTAATAGTT AATAGCGTAC TTCTT-----
NC_002645_HCoV 229E         ATAGAACAGT TTATGGCCCC ATTAAAAATG TGTACCACAT TTACCAATCA
NC_006577_HCoV HKU1         ACGA------ --ATAGGGTT GTTCATAGTA AACTTTTTCA TTTTT-----
NC_019843_MERS-CoV          ACGA------ --ATAGGGTT GTTCATAGTA AACTTTTTCA TTTTT-----
NC_006213_HCoV OC43         AGAC------ --ACTGTGTG GTATGTGGGG CAAATAATTT TTATA-----
NC_005831_HCoV NL63         GATG------ --ACAATGGT ATT-GTCCTC AATTCCATTT TATGG-----
FJ959407_SARS-CoV           AGAA------ --ACAGGTAC GTTAATAGTT AATAGCGTAC TTCTT-----
```

```
                           ....|....| ....|....| ....|....| ....|....| ....|....|
                                   210        220        230        240        250
NC_045512_SARS-CoV-2       ---------- ---TTTCTTG CTTTCGTGGT ATTCTTGCTA GTTACACTAG
NC_002645_HCoV 229E        TATATGCACA TAGACCCTTT CCCTAAACGA GTTATTGATT TCTAAACTAA
NC_006577_HCoV HKU1        ---------- ---ACCGTAG TATGTGCTAT AACACTCTTG GTGTGTATGG
NC_019843_MERS-CoV         ---------- ---ACCGTAG TATGTGCTAT AACACTCTTG GTGTGTATGG
NC_006213_HCoV OC43        ---------- ---GTTGCCA TTTGTTTATT GGTTACAATA GTTGTAGTGG
NC_005831_HCoV NL63        ---------- ---CTCCTTG TTATGATATT TTTCTTTGTG TTGGCAATGA
FJ959407_SARS-CoV          ---------- ---TTTCTTG CTTTCGTGGT ATTCTTGCTA GTCACACTAG

                           ....|....| ....|....| ....|....| ....|....| ....|....|
                                   260        270        280        290        300
NC_045512_SARS-CoV-2       CCATCCTTAC TGCGCTTCGA TTGTGTGCGT ACTG------ ---CTGCAAT
NC_002645_HCoV 229E        ACGACAATGT CAAATGACAA TTGTACGGGT GACA------ ---TTGTCAC
NC_006577_HCoV HKU1        CTTTCCTTAC GGCTACTAGA TTATGTGTGC AATGTATGAC AGGCTTCAAT
NC_019843_MERS-CoV         CTTTCCTTAC GGCTACTAGA TTATGTGTGC AATGTATGAC AGGCTTCAAT
NC_006213_HCoV OC43        CATTTTTGGC AACTTTTAAA TTGTGTATTC AACT------ ---TTGCGGT
NC_005831_HCoV NL63        CCTTTATTAA ACTGATTCAA TTGTGTTTTA CTTG------ ---TCATTAT
FJ959407_SARS-CoV          CCATCCTTAC TGCGCTTCGA TTGTGTGCGT ACTG------ ---CTGCAAT

                           ....|....| ...
                                   310
NC_045512_SARS-CoV-2       A--TTGTTAA CGT
NC_002645_HCoV 229E        C--CATTTGA AGA
NC_006577_HCoV HKU1        ACCCTGTTAG TTC
NC_019843_MERS-CoV         ACCCTGTTAG TTC
NC_006213_HCoV OC43        A--TGTGTAA TAC
NC_005831_HCoV NL63        T--TTTTTAG TAG
FJ959407_SARS-CoV          A--TTGTTAA CGT
```

Supplement S1.

Standard operation procedures (SOP) for SARS-CoV-2 detection in clinical sample by using COVID-19-LAMP

Materials required

1. QIAamp Viral RNA Mini Kit (QIAGEN, #52906, 250 reactions) or equivalent
2. Vacuum manifold (optional)
3. Materials required for viral nucleic acid extraction as recommended by the extraction kit protocol
4. 96-100% ACS grade ethanol
5. Warmstart Colorimetric LAMP 2X Master Mix (NEB, M1800S, 100 reactions)
6. Six primers (F3, B3, FIP, BIP, LoopF, LoopB)
7. Microcentrifuge (adjustable, up to 8000 × *g*)
8. Heat block with lid/heated lid or thermal cycler (for 60°C incubation)
9. Thermometers (for confirming temperature of heat block during incubation)
10. Adjustable pipettes (10, 20, 100, 200, 1000 µL)
11. Sterile, RNase-free pipette tips with aerosol barrier
12. PCR-grade water
13. 75% ethanol or 2% Virkon for disinfection

Biosafety requirements

1. All procedures should be performed in Biosafety Level 2 (BSL-2) laboratory, or in a separate ventilated compartment of a mobile/temporary diagnostic unit.
2. All procedures involving non-inactivated clinical samples should be performed inside a validated class II biosafety cabinet or a sample processing chamber with UV lamp in a mobile/temporary diagnostic unit if it is available.
3. Laboratory gown and gloves shall be worn at all procedures. Sleeves, disposable gloves and surgical mask shall be worn when non-inactivated specimens are being handled, in a BSL-2 laboratory with class II biosafety cabinet setting.
4. If a BSL-2 laboratory or class II biosafety cabinet is not available, for example in a mobile or temporary diagnostic unit, personal protection equipment (PPE) including N95 masks, safety googles, face shields, disposable lab gowns, gloves and hair covers should be worn before inactivation of the virus in the samples.
5. After completing all procedures, discard the PPE carefully inside the biosafety cabinet or a UV chamber (if it is available) and package and tie/seal them with plastic bags. Wash hands with soap/hand rub with 70-75% ethanol immediately for at least 30 seconds.

Sample requirements

Respiratory specimens: Nasopharyngeal swabs/aspirates, sputa/deep throat saliva, throat swabs in transport medium or viral inactivation collection tubes. Specimens should be kept at 2-4°C or extract within four hours.

Operation procedures

RNA extraction from specimens (based on QIAamp Viral RNA Mini Kit)

(Outside biosafety cabinet or sample processing UV chamber)

1. Prepare the lysis buffer as instructed by the manufacturer's protocol.
   (Add 5.6 µL of carrier RNA in each 0.56 mL of AVL buffer, mix by inverting the tube 5-10 times. DO NOT VORTEX.)
2. Aliquot appropriate amount of lysis buffer (560 µL of AVL buffer) into each screw cap tubes.
3. Transfer the screw cap tube (with lysis buffer) to biosafety cabinet/sample processing UV chamber.

(Inside biosafety cabinet/UV chamber)

4. Aliquot the samples into the screw cap tubes containing the lysis buffer (140 µL if using QIAamp Viral RNA Mini Kit).
5. Mix by pipetting up and down 5 times and inverting up and down 5 times. Incubate the mixture at room temperature for 2 minutes.

6. Disinfect the inactivated sample tubes with 2% Virkon/75% ethanol before taking out from biosafety cabinet/UV chamber.
7. Store remaining specimen bottles in a large plastic double bag, labelled with date of requested for temporary storage.
8. Disinfect the biosafety cabinet/UV chamber using 2% Virkon or 75% ethanol after use.

(Outside biosafety cabinet/UV chamber)

9. Spin down the lysate.
10. Add 560 μL of 96-100% ACS grade ethanol to the sample. Mix by vortexing/inverting and spinning down.
11. Add 630 μL of the mixture to column, spin at 8000 × *g* for 30 s (or using a vacuum manifold to pull down the samples). Repeat this step until all mixture has been loaded into column.
12. Discard the flow-through. Add 500 μL of AW1, spin at 6000 × *g* for 30 s (or using a vacuum manifold to pull down the washing solutions).
13. Discard the flow-through. Add 500 μL of AW2, spin at full speed for 30 s (or using a vacuum manifold to pull down the washing solutions).
14. Place the column into a new 2 mL collection tube, dry spin at full speed for 1 min (or using a vacuum manifold to pull down the washing solutions) to remove residual AW2.
15. Place column to a 1.5 mL tube. Add 60 μl of AVE buffer. Incubate at room temperature for 1 min. Spin at 8000 × *g* for 1 min to collect the RNA.
16. The extracted RNA tubes should be disinfected by 2% Virkon/75% ethanol before passing to the LAMP reaction room/compartment for safety concern.
17. The extracted RNA will be passed in an air-sealed UV pass box or in a clean secondary container to the LAMP reaction room/compartment.

(In LAMP reaction room/compartment)

Preparation of primer mix

18. Primer mix of six primers (F3, B3, FIP, BIP, LoopF, LoopB) with a final concentration of (F3/B3: 0.61 μM; FIP/BIP: 2.42 μM; LoopF/LoopB: 1.21 μM) in a total volume of 750 μL.

Preparation of COVID-19-LAMP reaction mix

19. For each 1.25 mL of Warmstart colorimetric LAMP 2× Mastermix, 750 μL of primer mix will be added to it.
20. Pre-aliquot each 20 μL of the reaction mix to a 0.5 mL tube. (store under −20°C for long-term storage or under 4°C for use within one day)
21. Label reaction tubes according to samples list.
22. Label positive and negative control tubes.

COVID-19-LAMP Reaction

21. Add 5 μl of RNA or controls to each LAMP reaction tube for final reaction volume of 25 μl.
22. Spin down the tubes if needed.
23. Place the colorimetric RT-LAMP reaction tubes in a 60°C heat block with lid/heated lid or thermal cycler for a maximum of 90 min. Thermometers should be used to confirm the temperature. Condensation can be minimized if heat block with heated lid or thermal cycler is used. If a heat block without lid is used, wrap the heat block with aluminum foil and utilize good thermal insulation materials such as polyurethane/polystyrene foam, cellulose, fiberglass and mineral wool as temporary lid.
24. Quick spin before result inspection to avoid condensation of solutions on top of tubes.
25. Inspect result only at 30 min, 60 min and 90 min to avoid temperature disturbance. (To avoid amplicon contamination, DO NOT open the LAMP reaction tubes after addition of RNA.)

Result visualization

26. If the color in the reaction is changed from pink to yellowish-orange or yellow at either 60 min or 90 min, it is regarded as a positive result, i.e. SARS-CoV-2 RNA is detected (see photo below).
At 60 min, if the color is changed from pink to orange or remained unchanged, it is

regarded as a negative result and should be incubate until 90 min.
At 90 min, if the color is still pink or orange, it is regarded as a negative result, i.e. SARS-CoV-2 RNA is NOT detected (see photo and guide below).

Negative Positive

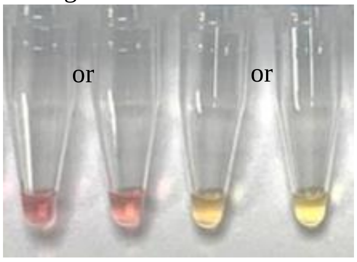

Result interpretation guide

	30 min	60 min	90 min	Interpretation
**Scenario 1**	Yellow	Yellow	Yellow	Positive
**Scenario 2**	Pink/Orange	Yellow	Yellow	Positive
**Scenario 3**	Pink/Orange	Pink/Orange	Yellow	Positive
**Scenario 4**	Pink	Pink	Pink/Orange	Negative
**Scenario 5 with negative control**	Yellow	Yellow	Yellow	Contamination, repeat
**Scenario 6 with positive control**	Pink	Pink	Pink	Repeat
**Positive control**	Yellow	Yellow	Yellow	Positive
**Negative control**	Pink	Pink	Pink	Negative

***** End of Document *****

References:

QIAamp® Viral RNA Mini Handbook January 2020 (2020). QIAGEN, Germany.

Laboratory Biosafety Guidance Related to Coronavirus Disease 2019 (COVID-19) Interim guidance 12 February 2020 (2020). World Health Organization.

Laboratory Biosafety Manual Third Edition (2004). World Health Organization, Geneva.

Biosafety in Microbiological and Biomedical Laboratories Fifth Edition (2009). Centers for Disease Control and Prevention, USA.

Version: 1.0, April 2020
